# The impact of human papillomavirus (HPV) status on functional outcomes and quality of life (QOL) after surgical treatment of oropharyngeal carcinoma with free-flap reconstruction

**DOI:** 10.1186/s40463-018-0301-z

**Published:** 2018-09-19

**Authors:** Hani Z. Marzouki, Vincent L. Biron, Peter T. Dziegielewski, Andrew Ma, Jason Vaz, Gabriela Constantinescu, Jeffrey Harris, Daniel O’Connell, Hadi Seikaly

**Affiliations:** 1 Department of Otolaryngology-Head and Neck Surgery, King Abdulaziz University, Jeddah, Saudi Arabia; 2 Division of Otolaryngology-Head and Neck Surgery, Department of Surgery, University of Alberta and Alberta Health Services, 1E4.34, WMC 8440 - 112 Street, Edmonton, AB T6G 2B7 Canada; 3 Department of Otolaryngology, University of Florida, Florida, USA

## Abstract

**Background:**

To determine the impact of Human Papillomavirus (HPV) status on speech, swallowing, and quality of life (QOL) outcomes after surgical treatment of oropharyngeal cancer (OPSCC).

**Methods:**

A retrospective review of a prospectively collected database of all patients with OPSCC diagnosed and treated from 1998 to 2009. Speech, swallowing, and quality of life data were gathered at 3 different evaluation points. HPV status was determined using p16 positivity as a surrogate marker. Univariate and multivariate statistical analyses were performed to identify whether p16 status is a significant predictor of functional outcome and QOL.

**Results:**

One hundred twelve patients with OPSCC and known p16 status were treated with primary surgery between 1998 and 2009, with mean age of 56 years. Out of those patients 63 (56%) were p16 positive. Speech intelligibility remained high at 1-year post operation (95.4%). Only 11.5% of the patients required a feeding tube at 1 year after surgery to maintain their daily caloric requirements and the risk of aspiration after surgery was not significant (*p* = 0.097). There was no statistically or clinically significant difference in speech, swallowing ability, swallowing safety and QOL outcomes between p16-positive and negative OPSCC.

**Conclusions:**

Surgically treated OPSCC patients demonstrate excellent swallowing function and can achieve excellent speech perception. P16 status may not be predictive of functional outcomes or QOL in surgically treated OPSCC.

## Background

Traditionally, tobacco and alcohol use have subscribed to be the primary risk factors for oropharyngeal squamous cell carcinoma (OPSCC). However, the past decade has seen a rapid increase in the incidence of human papillomavirus (HPV) related oropharyngeal cancers in developing countries [1–4]. Molecular and epidemiologic studies suggest that HPV-positive oropharyngeal cancers comprise a distinct disease entity that has a higher survival and better response to treatment [5–8]. Although survival is the fundamental goal in treating oropharyngeal cancer patients, their functional and quality of life (QOL) outcomes are becoming a primary concern of the patients and their health care providers. The issue of these outcomes after surgical treatment has received considerable critical attention.

The optimal treatment of advanced stage OPSCC has been a subject of debate for several years. In population based studies, the 5-year survival of oropharyngeal cancer patients has been shown to be better when treated with primary surgical resection compared to primary radiation treatment [9–12]. Tschiesner et al. (2012) investigated the functional and quality of life outcomes between primary radiation and major surgical resection with free flap reconstruction in advanced head and neck cancer patients and found no significant difference [13]. The past decade has seen a significant paradigm shift toward surgical treatment of OPSCC coinciding with the introduction of less invasive transoral approaches [14, 15]. Studies have reported excellent functions and quality of life after transoral surgery for oropharyngeal cancer patients [15–17]. In previous studies conducted by our group we have found that surgically treated oral and oropharyngeal cancer patients have excellent functional outcomes including speech intelligibility and safe efficient swallowing [18–21] but this was not investigated in relation to HPV/p16 status. Several studies have reported improved survival outcomes with HPV positive vs. negative OPSCC but there is a paucity of knowledge regarding the role of HPV positivity on functional outcomes and quality of life, particularly in surgically treated patients.

The aim of this study is to determine the impact of the p16 status on speech, swallowing and QOL outcomes in OPSCC patients treated with primary surgery.

## Methods

Prior to commencing the study, ethical clearance was sought from the University of Alberta Health Research and Ethics Board.

### Study design and patients

The study involved a retrospective review of a prospectively collected outcomes dataset. The initial sample consisted of 226 patients with OPSCC, treated and followed in the University of Alberta Hospital from 1998 to 2009. All of these patients were treated with major resection and free flap reconstruction. As part of our standard care, patients are assessed at the visit the Institute for Reconstructive Sciences in Medicine (iRSM) at the Misericordia Hospital in Edmonton, Alberta, Canada; at 3 different times for speech, swallowing and QOL assessments with a speech-language pathologist. A retrospective chart review of these patients was completed to determine the following parameters: age, gender, risk factors, site of lesion, TNM staging and speech, swallowing and QOL data.

### Subjects selection and withdrawal

#### Inclusion criteria

1. Adult patients (> 18 years).
2. Primary cancer of the oropharynx.
3. Primary treatment was surgical.
4. Available speech and swallowing outcomes data.
5. Available QOL data.

#### Exclusion criteria

1. Patients younger than 18 years.
2. Head and neck cancer other than the oropharynx.
3. Treated primarily with radiation therapy.
4. Speech and swallowing data not available.
5. QOL data not available.

### Functional assessment

Swallowing and speech functions and QOL were measured and prospectively recorded at 3 points in time: preoperatively, 6 months postoperative; and 1 year postoperative at the.

Head and Neck Surgery Functional Assessment Laboratory at the iRSM [22].

Objective functional outcomes were measured as follows:

#### Speech assessment

Single Word Intelligibility (SWI) and Sentence Intelligibility (SI) as determined by Naїve listener were measured using the standard Computerized Assessment of Intelligibility of Dysarthric Speech (CAIDS; Pro-Ed, Austin, TX) [22, 23].

#### Swallowing assessment

Swallowing was assessed in terms of ability (gastrostomy tube (g-tube) requirement rate) and safety (risk of aspiration) of swallowing. Swallowing ability was defined as complete independence from a g-tube to maintain their daily caloric requirements. Video Fluoroscopic Swallowing Studies (VFSS), using a standard Penetration-Aspiration Scale [24], were used to evaluate swallowing safety [22, 24] . Patients were divided into two groups according to the Penetration-Aspiration Scale (Aspiration group and No Aspiration group).

### Quality of life (QOL) assessment

QOL was measured using the European Organization for Research and Treatment of Cancer Head & Neck 35 Quality of Life Questionnaire (EORTC H&N 35). Subjects were asked to fill out the questionnaire to assess their QOL, which takes 20–30 min each time. Scores were scaled up to a maximum of 100. Minimal clinically important differences were approximated as described by others [25]**.**

### HPV status and P16 immunohistochemistry staining

**Fig. 1:**
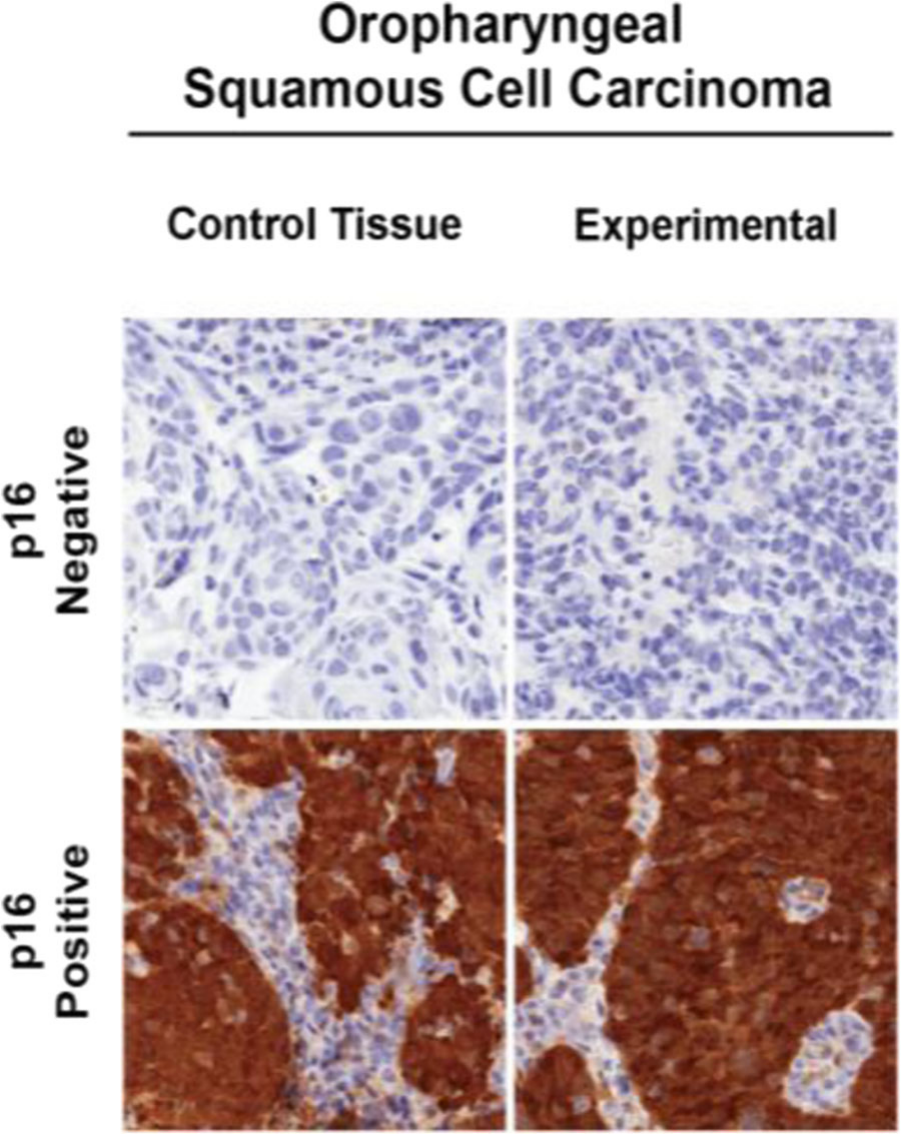
Histopathologic slide showing P16 positivity

Several different methods have been developed and introduced to identify HPV status. To date viral DNA amplification by the Polymerase Chain Reaction (PCR) is still the most sensitive test [26–28]. Immunohistochemistry (IHC) using p16 has been used as a surrogate marker of oncogenic HPV and found to have a significant concordance rate of more than 80% with PCR testing and this test is now widely available and accepted as a method of detecting HPV status, and has been used in many investigational studies [3, 10, 29–31]. HPV status of patients in this study was determined by p16 immunohostochemistry of formalin-fixed paraffin embedded (FFPE) tumors as previously reported [10]. Arrays were subjected to both a standard hematoxylin and eosin (H&E) staining and a p16INK4a mouse monoclonal antibody (p16) (Fig. 1). P16 positivity was digitally determined using AQUAnalysis software (HistoRx, Inc. Branford, Connecticut) as previously described [10–12, 32].

### Data analysis

Data management and analysis was performed using SPSS version 20. A univariate comparison between the groups was made using Mann-Whitney Test for the continuous data and Chi square test for the categorical data. The Wilcoxon non-parametric test was also used to compare the data across the time periods and the Friedman test was used for the statistical analysis of QOL. The mean scores for word and sentence intelligibility were compared using linear regression analysis. A multivariate analysis of the different variables with potential effect on the rate of g-tube requirement was done using a logistic regression analysis. Differences between groups were deemed to be statistically significant with a *p* value < 0.05.

## Results

### Patient demographics and clinical characteristics

Table 1 summarizes the demographics and clinical characteristics of the 112 patients that have met the inclusion/exclusion criteria and included in this study. Similar to previous studies, the majority of OPSCC in this cohort were relatively young (mean age 56 years), p16 positive males. Most patients also presented with advanced stage disease as according the AJCC 7th edition (Table 2).

**Table 1:** Demographics and clinical characteristics

Characteristic	No. (%)
*N*	112
Age (yrs)	56
Gender	
Male	92 (82%)
Female	20 (18%)
p16 status	
P16 Positive (+)	63 (56%)
P16 Negative (−)	49 (44%)

**Table 2:** Primary Tumor Stages

	N0	N1	N2a	N2b	N2c	N3	TOTAL
T1	1 (1%)	2 (2%)	4 (3.5%)	2 (2%)	0 (0%)	2 (2%)	11 (10%)
T2	1 (1%)	5 (4.5%)	7 (6.25%)	10 (9%)	2 (2%)	5 (4.5%)	30 (27%)
T3	13 (12%)	6 (5.4%)	7 (6.25%)	18 (16%)	3 (2.7%)	2 (2%)	49 (44%)
T4	1 (1%)	5 (4.5%)	3 (2.7%)	5 (4.5%)	7 (6.25%)	1 (1%)	22 (19%)
TOTAL	16 (14%)	18 (16%)	21 (19%)	35 (31%)	12 (11%)	10 (9%)	112

### Functional outcome results

#### Speech function

##### Single word and sentence intelligibilities

The results in Tables 3 and 4 showed an 85% postoperative mean score for word intelligibility. On the other hand, showed an excellent postoperative sentence intelligibility of 95.4%. Overall, p16 status did not show any statistical significant effect on speech intelligibility.

**Table 3:** Single word intelligibility

Intelligibility	Pre-Op	6 Mo Post-Op	12 Mo Post-Op	Δ	*p*-value
All	94.4%	85.5%	85%	9.4%	< 0.001
P16 -	92%	85.1%	84.8%	7.2%	.003
P16 +	95.7%	84.4%	85.1%	10.6%	< 0.001
*p*-value (p16)	0.248	0.418	0.795

**Table 4:** sentence intelligibility

Intelligibility	Pre-Op	6 Mo Post-Op	12 Mo Post-Op	Δ	*p*-value
Sentence	98.7%	94%	95.4%	3.3%	< 0.001
P16-	98.2%	92.1%	94.6%	3.6%	.053
P16+	99%	95%	95.8%	3.2%	< 0.001
*p*-value (p16)	0.144	0.207	0.897		

A Multivariable analysis using linear-regression was used to assess the effect of p16 status, age, stage and the percentage of base of tongue and soft palate resection on intelligibility. This analysis revealed a positive correlation between patients’ age and the percentage of base of tongue resection on both single word and sentence intelligibilities with *p*-values less than 0.05. There were no significant differences in intelligibilities in relation to p16 status (Tables 5 and 6).

**Table 5:** Single word intelligibility – Multivariable analysis

Variables	*p-*Value	95*%* confidence interval
P16	0.22	−5.74- 13.74
Increase in AGE	0.04	0.08–0.80
T Stage	0.54	−12.16- 11.89

**Table 6:** Sentence intelligibility – Multivariable analysis

Variables	*P-*Value	95*%* confidence interval
P16	0.86	−3.95- 5.29
Increase in AGE	0.01	0.05–0.39
T Stage	0.56	−7.58- 3.83

#### Swallowing function

##### Swallowing ability

Only 13% of patients required a g-tube 12–months after surgery to maintain their daily caloric requirements and there was no statistical significant difference in the rate of g-tube dependency in relation to p16 status (Table 7). The results, as shown in Table 8, indicate that the percentage of base of tongue resection has a significant association with the rate of g-tube dependency.

**Table 7:** Swallowing Ability – The rate of G-Tube requirement

	ALL (% with g-tube)	P16- (% with g-tube)	P16 + (% with g-tube)	HPV+/HPV- (*p*-Value)
Pre OP	2	6	0	0.068
6 M Post OP	29.6	41	23	0.084
12 M Post OP	11.5	21	9	0.172

**Table 8:** The rate of G-Tube requirement – Multivariable analysis

Variables	*P-*Value
p16	0.23
AGE	0.9
T Stage	0.42
% BOT	0.01
% SP	0.37

Logistic regression analysis

*BOT* Base of Tongue, *SP* Soft Palate

##### Swallowing safety

Overall, swallowing was found to be safe post-operatively and there was no increase in the risk of aspiration was detected in relation to p16 status. None of these differences were statistically significant (Table 9).

**Table 9:** Swallowing Safety (the risk (%) of aspiration

	All	P16-	P16+	*p*-Value
Pre OP	0%	0%	0%	
6 M Post Op	11.5%	17.4%	8%	0.26
12 M Post Op	12%	17.6%	9.4%	0.4
*p*-Value	0.097	0.223	0.368	

Using Video Fluoroscopic Swallowing Study (VFSS) to Measure the risk of aspiration according to a standard Penetration-Aspiration Scale

### Quality of life (QOL) results

Patients were divided into three groups according to the change in their QOL scores. Change (Δ) in mean score:

1. Mild Δ = 5–10
2. Moderate Δ = 10–20
3. Severe Δ ≥ 20

What has been considered as clinically significant was mean score difference of more than 10 [33–35].

#### Overall quality of life

**Fig. 2:**
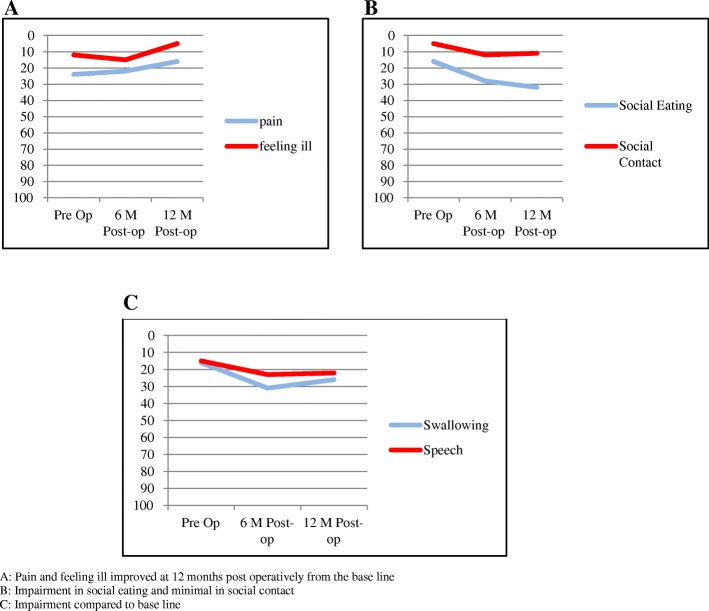
Overall Quality of Life (QOL) Domains: **a**: Pain and feeling ill improved at 12 months post operatively from the base line. **b**: Impairment in social eating and minimal in social contact. **c**: Impairment compared to base line

From this data, we can see that the overall QOL scores postop and preop was excellent (Tables 10 and 11), although the difference was statistically significant, but the differences in the mean scores was not clinically significant (Δ in mean score < 10). Figure 2 presents an overview of the QOL outcomes by time frame and the main QOL domains.

**Table 10:** Quality of Life (QOL) mean Scores

	Pre-Op Mean score (SD)	6 months Mean score (SD)	12 months Mean score (SD)	Δ	*P-* Value
pain	24 (23)	22 (17.6)	16 (17.3)	−8	0.02
swallowing	16 (23)	31 (22.6)	26 (22.2)	10	< 0.01
senses	14 (21.2)	26 (20.7)	21 (23.2)	7	< 0.01
speech	15 (18.3)	23 (18.7)	22 (19.4)	7	0.03
social eating	16 (25)	28 (22.3)	32 (24.3)	16	< 0.01
social contact	5 (12.4)	12 (18)	11 (17.6)	6	0.004
sexuality	16 (28)	23 (28.1)	18 (27.7)	2	0.02
teeth	10 (22)	14 (21.1)	24 (32)	14	0.07
mouth open	20 (33)	39 (33)	42 (36.1)	22	< 0.01
coughing	30 (24.1)	40 (28)	34 (23.7)	4	0.03
felt ill	12 (21.3)	15 (24)	5 (15.5)	−7	0.002

**Table 11:** Overall Quality of Life (QOL) Scores

	*N*	Mean EORTC H&N 35 score (SD)
Pre-Op	85	19 (14.14)
6 M Post-p	68	32 (13.47)
12 M Post-p	68	27 (12.54)
Change (Δ)	17	8 (*p <* 0.01)

*Δ = 8 (< 10) not clinically significant*

#### Quality of life *(QOL) and p16*

When comparing different domains of QOL and p16 status it was found that p16 positive patients had improved social eating and sexuality compared to p16 negative patients (clinically and statistically significant) (Table 12). There was no clinical significant difference in the overall QOL in relation to p16 status (Table 12).

**Table 12:** p16 and Quality of Life (QOL) Scores

		**HPV - Mean score (SD)**	**HPV + Mean score (SD)**	**Δ**	***P-*** **Value**
**pain**		18 (17.34)	16 (17.37)	- 2	0.58
**swallowing**		30 (24.22)	27 (21.33)	- 3	0.63
**senses**		16 (19.77)	24 (24.54)	8	0.24
**speech**		21 (17.78)	23 (20.42)	2	0.75
**social eating**		43 (31.53)	26 (17.29)	**- 17**	**0.04**
**social contact**		11 (17.71)	11 (17.82)	0	0.99
**sexuality**		28 (35.87)	16.5 (22.69)	**- 11.5**	0.19
**teeth**		23 (32.46)	24 (32.1)	1	0.84
**mouth open**		45 (38.41)	40 (35.25)	- 5	0.64
**coughing**		35 (23.52)	34 (24.09)	- 1	0.89
**felt ill**		1.5 (6.95)	7 (18.26)	5.5	0.41
	**N**	**P16 - Mean score (SD)**	**P16 + Mean score (SD)**	**Δ**	**P-Value**
**Pre-Op**	82	22.5 (15.6)	13 (12.1)	- 9.5	**0.005**
**6 M Post-p**	65	33.5 (16)	29 (12)	- 4.5	0.24
**12 M Post-p**	65	28.5 (10.5)	26 (13.4)	- 2.5	0.45

Significant *p*-value are highlited in bold

## Discussion

The current literature suggests that surgical treatment of advanced oropharyngeal cancer is associated with poor QOL and functional outcomes. Results of our study suggest the contrary; these patients achieved excellent swallowing abilities with only 13% of the patients required G-Tube at 12 months post treatment, which is comparable to patients that underwent TORS [16, 36] and speech perception, with sentence recognition by naïve listeners that was near perfect post-surgery. Although word intelligibility is a sensitive test for speech outcome, it is not clinically important. The results shown in Tables 3 and 4 indicate that the mean score for postoperative word intelligibility dropped on the order of 9%. On the other hand, sentence intelligibility that is more clinically important as it represents listener appreciation of contextual speech showed only a 3% drop in intelligibility after surgery. Overall, p16 status did not affect Sentence/contextual speech. Contextual speech was still well understood in both p16 positive and p16 negative patients with no significant differences. Advanced stage OPSCC patients treated with primary surgical approaches have also been shown to have better survival outcomes, especially those in the high-risk groups (HPV negative and smokers) [10, 37] . Primary surgery for OPSCC can therefore provide excellent oncologic and functional outcomes.

HPV positive OPSCC is known to have excellent treatment response with high survival rates shown to extend beyond 5 years [38–41]. With the rising population of HPV-OPSCC survivors, investigating the functional outcomes and QOL of these patients is of utmost importance. To date, few studies have reported an association between HPV status and functional outcomes and/or QOL in OPSCC [25]. A study of 177 OPSCC patients (45% p16 +) reported higher University of Washington (UW) QOL scores in p16 positive patients pre- and post-treatment [42]. Another study using showed higher pre-treatment UW QOL scores in HPV positive vs negative OPSCC, however this association did not persist beyond 1 year post-treatment [43]. In a multicenter study of 48 patients with OPSCC (39% HPV+), no significant differences in EORTC QOL surveys between HPV positive and negative patients [44]. When comparing surgical versus non-surgical treatments, these studies did not show any differences in QOL measurements. In a retrospective analysis of OPSCC patients treated with CRT, p16 positive patients had better baseline QOL scores but demonstrated a greater reduction in QOL post-treatment [45]. An independent study comparing TORS and CRT treatments showed that patients treated with primary TORS approaches had significantly better saliva-related QOL [46]. The present study was designed to determine the association of p16 status on the functional outcomes and QOL of surgically treated OPSCC with open surgery and free flap reconstruction that is more. Our patient cohort was unique with a large number of surgically treated patients, however similar to other studies, we did not find significant differences between p16 positive and negative OPSCCs. Regardless of p16 status, surgically treated OPSCC patients had excellent QOL, speech and swallowing outcomes.

This study was limited by a lack of reliable tobacco use history available as part of our retrospective analysis. Only surgically treated patients were included in this study and therefore comparisons cannot be made with patients who received CRT or RT alone. In addition, a single instrument was used to measure QOL, however, the EORTC H&N 35 has been validated and is one of the most widely accepted QOL questionnaires for OPSCC [25, 44, 47].

## Conclusion

This study suggests that primary surgery for OPSCC results in excellent QOL and functional outcomes that are not associated with p16 status. Further prospective studies comparing surgical and non-surgically treated patients according to HPV/p16 status may be beneficial in predicting and optimizing outcomes.

## Abbreviations

ACR: Alberta Cancer Registry
BOT: Base of tongue
CRT: Chemoradiation
EORTC: European Organization for Research and Treatment of Cancer
G-tube: Gastrostomy tube
HPV: Human papillomavirus
HPV-OPSCC: Human papillomavirus-related oropharyngeal squamous cell carcinoma
IHC: Immunohistochemistry
iRSM: Institute for Reconstructive Sciences in Medicine
NCCN: National Comprehensive Cancer Network
OPSCC: Oropharyngeal squamous cell carcinoma
PCR: Polymerase chain reaction
QOL: Quality of life
RT: Radiation therapy
SD: Standard deviation
SI: Sentence intelligibility
SP: Soft palate
SWI: Single word intelligibility
VFSS: Video fluoroscopic swallowing study

## References

[1] Kreimer AR, Clifford GM, Boyle P, Franceschi S (2005). Human papillomavirus types in head and neck squamous cell carcinomas worldwide: a systematic review. Cancer Epidemiol Prev Biomarkers.

[2] Näsman A, Attner P, Hammarstedt L, Du J, Eriksson M, Giraud G (2009). Incidence of human papillomavirus (HPV) positive tonsillar carcinoma in Stockholm, Sweden: an epidemic of viral-induced carcinoma?. Int J Cancer.

[3] Hayes DN, Van Waes C, Seiwert TY (2015). Genetic landscape of human papillomavirus-associated head and neck Cancer and comparison to tobacco-related tumors. J Clin Oncol.

[4] Gillison ML, Chaturvedi AK, Anderson WF, Fakhry C (2015). Epidemiology of human papillomavirus-positive head and neck squamous cell carcinoma. Proc Am Soc Clin Oncol.

[5] Lindsay C, Seikaly H, Biron VL (2017). Epigenetics of oropharyngeal squamous cell carcinoma: opportunities for novel chemotherapeutic targets. J Otolaryngol Head Neck Surg.

[6] Gillison ML, Gillison ML, D’souza G, Westra W, Westra W (2008). Distinct Risk Factor Profiles for Human Papillomavirus Type 16-Positive and Human Papillomavirus Type 16-Negative Head and Neck Cancers. J Natl Cancer Inst.

[7] Gillison ML, Shah KV (2001). Human papillomavirus-associated head and neck squamous cell carcinoma: mounting evidence for an etiologic role for human papillomavirus in a subset of head and neck cancers. Curr Opin Oncol.

[8] Lydiatt WM, Patel SG, O’Sullivan B, Brandwein MS, Ridge JA, Migliacci JC (2017). Head and Neck cancers-major changes in the American Joint Committee on cancer eighth edition cancer staging manual. CA Cancer J Clin.

[9] O’Connell D, Seikaly H, Murphy R, Fung C, Cooper T, Knox A (2013). Primary surgery versus chemoradiotherapy for advanced oropharyngeal cancers: a longitudinal population study. J Otolaryngol Head Neck Surg.

[10] Seikaly H, Biron VL, Zhang H, O’Connell DA, Côté DWJ, Ansari K (2016). Role of primary surgery in the treatment of advanced oropharyngeal cancer. Head Neck.

[11] Cooper T, Biron VL, Adam B, Klimowicz AC, Puttagunta L, Seikaly H (2015). Association of keratinization with 5-year disease-specific survival in oropharyngeal squamous cell carcinoma. JAMA Otolaryngol Head Neck Surg.

[12] Cooper T, Biron V, Adam B, Klimowicz AC, Puttagunta L, Seikaly H (2013). Prognostic utility of basaloid differentiation in oropharyngeal cancer. J Otolaryngol Head Neck Surg.

[13] Tschiesner U, Schuster L, Strieth S, Harréus U (2012). Functional outcome in patients with advanced head and neck cancer: surgery and reconstruction with free flaps versus primary radiochemotherapy. Eur Arch Otorhinolaryngol.

[14] Chauhan P, Byrne H, Taylor E, Sheahan P (2015). Oncological and functional outcomes of transoral surgery for the treatment of oropharyngeal cancer. Ir J Med Sci.

[15] Lörincz BB, Busch C-J, Möckelmann N, Knecht R (2015). Feasibility and safety of transoral robotic surgery (TORS) for early hypopharyngeal cancer: a subset analysis of the Hamburg University TORS-trial. Eur Arch Otorhinolaryngol.

[16] Dziegielewski PT, Teknos TN, Durmus K, Old M, Agrawal A, Kakarala K (2013). Transoral robotic surgery for oropharyngeal cancer: long-term quality of life and functional outcomes. JAMA Otolaryngol Head Neck Surg.

[17] Biron VL, O’Connell DA, Barber B, Clark JM, Andrews C, Jeffery CC (2017). Transoral robotic surgery with radial forearm free flap reconstruction: case control analysis. J Otolaryngol Head Neck Surg.

[18] Brown L, Rieger JM, Harris J, Seikaly H (2010). A longitudinal study of functional outcomes after surgical resection and microvascular reconstruction for oral cancer: tongue mobility and swallowing function. J Oral Maxillofac Surg.

[19] Seikaly H, Rieger J, Zalmanowitz J, Tang JL, Alkahtani K, Ansari K (2008). Functional soft palate reconstruction: a comprehensive surgical approach. Head Neck.

[20] Seikaly H, Rieger J, O’Connell D, Ansari K, Alqahtani K, Harris J (2009). Beavertail modification of the radial forearm free flap in base of tongue reconstruction: technique and functional outcomes. Head Neck.

[21] O’Connell DA, Rieger J, Harris JR, Dziegielewski P, Zalmanowitz J, Sytsanko A (2008). Swallowing function in patients with base of tongue cancers treated with primary surgery and reconstructed with a modified radial forearm free flap. Arch. Otolaryngol. Head Neck Surg.

[22] Rieger JM, Zalmanowitz JG, Li SYY, Sytsanko A, Harris J, Williams D (2007). Functional outcomes after surgical reconstruction of the base of tongue using the radial forearm free flap in patients with oropharyngeal carcinoma. Head Neck.

[23] Yorkston KM, Beukelman DR (1981). Communication efficiency of dysarthric speakers as measured by sentence intelligibility and speaking rate. J Speech Hear Disord.

[24] Robbins J, Coyle J, Rosenbek J, Roecker E, Wood J (1999). Differentiation of normal and abnormal airway protection during swallowing using the penetration-aspiration scale. Dysphagia.

[25] Høxbroe Michaelsen S, Grønhøj C, Høxbroe Michaelsen J, Friborg J, von Buchwald C (2017). Quality of life in survivors of oropharyngeal cancer: a systematic review and meta-analysis of 1366 patients. Eur J Cancer.

[26] McKaig RG, Baric RS, Olshan AF (1998). Human papillomavirus and head and neck cancer: epidemiology and molecular biology. Head Neck.

[27] Isaac A, Kostiuk M, Zhang H, Lindsay C, Makki F, O’Connell DA (2017). Ultrasensitive detection of oncogenic human papillomavirus in oropharyngeal tissue swabs. J Otolaryngol Head Neck Surg.

[28] Biron VL, Kostiuk M, Isaac A, Puttagunta L, O’Connell DA, Harris J (2016). Detection of human papillomavirus type 16 in oropharyngeal squamous cell carcinoma using droplet digital polymerase chain reaction. Cancer.

[29] Thomas J, Primeaux T (2012). Is p16 immunohistochemistry a more cost-effective method for identification of human papilloma virus-associated head and neck squamous cell carcinoma?. Ann Diagn Pathol.

[30] Licitra L, Perrone F, Bossi P, Suardi S, Mariani L, Artusi R (2006). High-Risk Human Papillomavirus Affects Prognosis in Patients With Surgically Treated Oropharyngeal Squamous Cell Carcinoma. J Clin Oncol.

[31] Chung CH, Zhang Q, Kong CS, Harris J, Fertig EJ, Harari PM (2014). p16 protein expression and human papillomavirus status as prognostic biomarkers of nonoropharyngeal head and neck squamous cell carcinoma. Proc Am Soc Clin Oncol.

[32] Xu CC, Biron VL, Puttagunta L, Seikaly H (2013). HPV status and second primary tumours in oropharyngeal squamous cell carcinoma. J Otolaryngol Head Neck Surg.

[33] King MT (1996). The interpretation of scores from the EORTC quality of life questionnaire QLQ-C30. Qual Life Res.

[34] Osoba D, Rodrigues G, Myles J, Zee B, Pater J (1998). Interpreting the significance of changes in health-related quality-of-life scores. J Clin Oncol.

[35] Fang F-M, Chien C-Y, Kuo S-C, Chiu H-C, Wang C-J (2004). Changes in quality of life of head-and-neck cancer patients following postoperative radiotherapy. Acta Oncol.

[36] Hirshoren N, Ruskin O, Fua T, Kleid S, Magarey M, Dixon B (2016). Transoral robotic surgery: implementation as a tool in head and neck surgery - a single-Centre Australian experience. ANZ J Surg.

[37] Broglie MA, Stoeckli SJ, Sauter R, Pasche P, Reinhard A, de Leval L (2017). Impact of human papillomavirus on outcome in patients with oropharyngeal cancer treated with primary surgery. Head Neck.

[38] Dale OT, Han C, Burgess CA, Eves S, Harris CE, White PL (2015). Long-term functional outcomes in surgically treated patients with oropharyngeal cancer. Laryngoscope.

[39] Fakhry C, Andersen KK, Eisele DW, Gillison ML (2015). Oropharyngeal cancer survivorship in Denmark, 1977-2012. Oral Oncol.

[40] Patel MA, Blackford AL, Rettig EM, Richmon JD, Eisele DW, Fakhry C (2016). Rising population of survivors of oral squamous cell cancer in the United States. Cancer.

[41] Larsen CG, Jensen DH, Carlander A-LF, Kiss K, Andersen L, Olsen CH (2016). Novel nomograms for survival and progression in HPV+ and HPV- oropharyngeal cancer: a population-based study of 1,542 consecutive patients. Oncotarget.

[42] Maxwell JH, Mehta V, Wang H, Cunningham D, Duvvuri U, Kim S (2014). Quality of life in head and neck cancer patients: impact of HPV and primary treatment modality. Laryngoscope.

[43] Sharma A, Méndez E, Yueh B, Lohavanichbutr P, Houck J, Doody DR (2012). Human papillomavirus-positive oral cavity and oropharyngeal cancer patients do not have better quality-of-life trajectories. Otolaryngol Head Neck Surg.

[44] Spinato G, Stellin M, Azzarello G, Bonazza D, Zanconati F, Politi D (2017). Multicenter research into the quality of life of patients with advanced oropharyngeal carcinoma with long-term survival associated with human papilloma virus. Oncol Lett.

[45] Ringash J, Fisher R, Peters L, Trotti A, O’Sullivan B, Corry J (2017). Effect of p16 Status on the Quality-of-Life Experience During Chemoradiation for Locally Advanced Oropharyngeal Cancer: A Substudy of Randomized Trial Trans-Tasman Radiation Oncology Group (TROG) 02.02 (HeadSTART). Int J Radiat Oncol Biol Phys.

[46] Ling DC, Chapman BV, Kim J, Choby GW, Kabolizadeh P, Clump DA (2016). Oncologic outcomes and patient-reported quality of life in patients with oropharyngeal squamous cell carcinoma treated with definitive transoral robotic surgery versus definitive chemoradiation. Oral Oncol.

[47] Broglie MA, Soltermann A, Haile SR, Röösli C, Huber GF, Schmid S (2013). Quality of life of oropharyngeal cancer patients with respect to treatment strategy and p16-positivity. Laryngoscope.

